# Extraction of *Lilium davidii var. unicolor* Planting Information Based on Deep Learning and Multi-Source Data

**DOI:** 10.3390/s24051543

**Published:** 2024-02-28

**Authors:** Yinfang Shi, Puhan Zhang, Zhaoyang Wang

**Affiliations:** 1 College of Geography and Environmental Science, Northwest Normal University, Lanzhou 730070, China; 2021222926@nwnu.edu.cn (P.Z.);; 2 Key Laboratory of Resource Environment and Sustainable Development of Oasis, Lanzhou 730070, China

**Keywords:** *Lilium davidii var. unicolor*, remote sensing extraction, deep learning, multi-source data

## Abstract

Accurate extraction of crop acreage is an important element of digital agriculture. This study uses Sentinel-2A, Sentinel-1, and DEM as data sources to construct a multidimensional feature dataset encompassing spectral features, vegetation index, texture features, terrain features, and radar features. The Relief-F algorithm is applied for feature selection to identify the optimal feature dataset. And the combination of deep learning and the random forest (RF) classification method is utilized to identify lilies in Qilihe District and Yuzhong County of Lanzhou City, obtain their planting structure, and analyze their spatial distribution characteristics in Gansu Province. The findings indicate that terrain features significantly contribute to ground object classification, with the highest classification accuracy when the number of features in the feature dataset is 36. The precision of the deep learning classification method exceeds that of RF, with an overall classification accuracy and kappa coefficient of 95.9% and 0.934, respectively. The Lanzhou lily planting area is 137.24 km^2^, and it primarily presents a concentrated and contiguous distribution feature. The study’s findings can serve as a solid scientific foundation for Lanzhou City’s lily planting structure adjustment and optimization and a basis of data for local lily yield forecasting, development, and application.

## 1. Introduction

Agriculture constitutes the lifeblood of a nation, and precise information regarding crop planting structure is imperative for assessing crop yields. With the evolution of computer-related technologies, remote sensing technology is pivotal in extracting crop planting structures due to its advantages, including wide coverage, high efficiency, and cost-effectiveness [1]. Consequently, the adjustment of local crop planting structures, crop management, and the sustainable development of agriculture significantly benefit from utilizing remote sensing technologies to extract crop planting structure information [2].

Currently, research by both domestic and foreign scholars aimed at identifying and extracting different crop planting areas predominantly relies on medium spatial resolution optical remote sensing images such as Landsat and MODIS [3,4]. However, the limitations of sensor performance render it challenging to ensure classification accuracy in areas with complex terrain [5]. Sentinel-2 imagery offers a novel data source for crop identification, featuring advantages like high spatial resolution, multiple spectral bands, and dual-satellite synchronous operation. In a study by Han et al. [6], the spectral characteristics and vegetation index information of Sentinel-2A images were employed to extract rapeseed planting areas, revealing that, in comparison with Landsat-8, Sentinel-2A images exhibit superior precision in extracting crop distribution information within complex planting areas. Similarly, Tian et al. [7] utilized Sentinel-2A images to delineate extensive winter wheat planting areas, emphasizing the pivotal role of its red-edge band in extracting winter wheat planting information. While optical remote sensing technology is well established, its practical applications are susceptible to the effects of time and weather conditions [8], leading to the diminished classification accuracy of ground objects. In contrast, radar images provided by the Sentinel-1 satellite operate continuously, overcoming temporal constraints and capturing ground feature information distinct from optical images [9,10]. The synergistic combination of optical and radar images maximizes their respective benefits, enhancing the ability to identify ground objects and improving information extraction accuracy. Notably, scholars [11,12,13,14] have validated that the integration of Sentinel-2 and Sentinel-1 images yields higher classification accuracy in surface information extraction, including wetland and agricultural land. Additionally, Zhang Hao et al. [15] incorporated topographic data into the western region of the Loess Plateau to obtain relatively precise information about abandoned land, underscoring the importance of topographic data in classifying areas with complex topography.

In recent years, deep learning has emerged as a focal point in remote sensing image classification research. Its superior learning and generalization capabilities, particularly in intricate feature extraction, surpass traditional machine learning methods, leading to heightened classification accuracy [16,17]. Research indicates that the application of deep learning to fine crop classification has yielded promising results. For instance, Liu et al. [18] employed the convolutional neural network method on Sentinel-2 remote sensing images to identify primary crops in Yuanyang County, Henan Province, achieving a classification accuracy of 96.39%. Similarly, Li et al. [19] compared and analyzed the extraction effect of five deep learning models in fine crop distribution applications based on Sentinel-2 multi-temporal remote sensing images, revealing that all five models yielded accuracy rates exceeding 90%. Although deep learning methods have achieved high accuracy on a single data source, combining deep learning classification methods with multi-source data for crop information extraction has yet to be thoroughly investigated. Therefore, this paper aims to explore how to combine deep learning methods with multi-source data to improve the accuracy and reliability of crop information extraction.

To address the above issues, we used the U-Net architecture in the deep learning method based on Sentinel-2A, Sentinel-1, and DEM multi-source image data, along with the best index, spectrum, texture, terrain, and radar feature dataset selected by the Relief-F algorithm, to identify lilies in Qilihe District and Yuzhong County of Lanzhou City, obtain their planting structure, and analyze their spatial distribution characteristics. In addition, a comparative analysis was conducted between the random forest classification method and the deep learning classification method. This study provides basic data support for planting structure adjustment, growth monitoring, yield estimation, and pest and disease monitoring of lilies, which helps to achieve sustainable agricultural development.

## 2. Overview and Data of the Study Area

### 2.1. Overview of the Study Area

The research area is located in Qilihe District and Yuzhong County, Lanzhou City, Gansu Province. Qilihe District is situated between 35°50′25″ N and 36°06′09″ N and between 103°36′43″ E and 103°54′28″ E, with a width of 21 km from east to west and a length of 33 km from north to south, covering a total area of 397.25 km^2^; Yuzhong County is situated between 35°34′20′′ N and 36°26′30′′ N and between 103°49′15′′ E and 104°34′40′′ E, and has a width of 66 km east to west and a length of 96 km north to south, covering a total area of 3301.64 km^2^. Both regions fall within a temperate semi-arid climate zone characterized by an average annual temperature of 9.3 °C and annual precipitation of 324.8 mm. The soil is not only loose and fertile but also has an exceptionally rich microbiological community, including various types of bacteria, fungi, and protozoa, rendering them ideal lily planting bases in Gansu Province.

**Figure 1:**
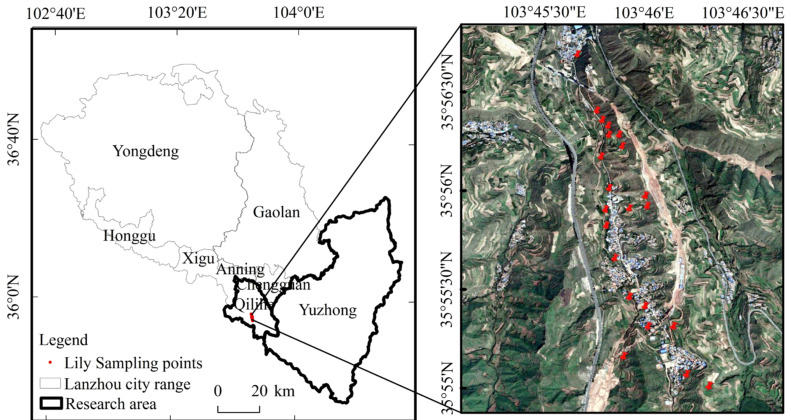
Schematic diagram of study area location and sampling points.

The “Lanzhou Lily” is a product of China’s National Geographical Indication, and the research area covers Xiguoyuan Town, Huangyu Township, Weiling Township in Qilihe District, Yinshan Township, Shanghuacha Township, and Yuanzicha Township in Yuzhong County. Lilies typically emerge in late April, flower from late June to early August; the stems and leaves on the ground wither gradually in September, and lilies hibernate in late October. After 2 years of lily planting, the lily needs to be re-transplanted for another 3 years, making the period from planting to harvesting 5 to 6 years. The ground data of this study comes from field experiments and survey-visit data in Xiguoyuan Town, Qilihe District in September 2021. This study’s ground data came from field investigations and survey-visit data in September 2021 in Xiguoyuan Town, Qilihe District, where a total of 22 lily sample points were collected using hand-held GPS, and the crop’s phenological period, planting mode, acreage, and fertilizer application, among other things, were recorded. The following Figure 1 is the spatial distribution of the sampling points in the study area.

### 2.2. Data

Sentinel-1 is equipped with a synthetic aperture radar sensor, endowing it with the capability to penetrate clouds and fog. Sentinel-2 covers 13 spectral bands within a width of 290 km, featuring a revisit period of 10 days, with the A and B satellites providing a complementary revisit duration of 5 days. The lily has luxuriant stems and leaves and high vegetation coverage during the flowering period, which is useful for extracting crop spatial distribution and planting information. Therefore, three Sentinel-2A L2A remote sensing images and two GRD-level Sentinel-1 images with wide mode (IW) were selected from early August, and the polarization method was VV + VH dual polarization to extract the planting structure of lilies. The image data were downloaded from the Copernicus Open Access Hub (https://scihub.copernicus.eu/dhus/#/home) (accessed on 7 and 9 August 2021), and the Sentinel-1 image was preprocessed for orbit correction, thermal noise removal, radiometric calibration, and terrain correction using the SNAP (version number: 9.0) software from the European Space Agency to obtain VV and VH backscatter coefficient maps. The nearest-neighbor method [20] was adopted to resample the Sentinel-1/2 image to 10 m for cropping and embedding. DEM data from GDEMV3 with a resolution of 30 m from the Geospatial Data Cloud Platforms (https://www.gscloud.cn) (accessed on 7 August 2021) were resampled to 10 m for slope, aspect, and elevation extraction.

In this study, we classified ground objects into five categories based on Google Earth 18-level high-resolution image data and field survey data, taking into account the texture and phenological properties of the image. These categories are artificial land, natural land, other crops, bare soil, and lilies. Artificial surfaces encompass man-made structures such as buildings, roads, and greenhouses, while natural surfaces refer to natural elements like woodlands and mountains. Additionally, other crops, such as potatoes and corn, are also considered. Existing research results have indicated that when the number of training samples reaches 10–30 times the number of classification features and the ratio of training samples to verification samples is 7:3, classification accuracy improves [21]. Therefore, 1177 training samples and 507 verification samples were uniformly and randomly selected in the study area using Google Earth and ENVI (version number: 7.3.2.5776) software, and their classification and quantity are displayed in Table 1. At the same time, this study generated a total of nine labeled raster images for deep learning model training.

## 3. Methods

### 3.1. Technical Process

**Figure 2:**
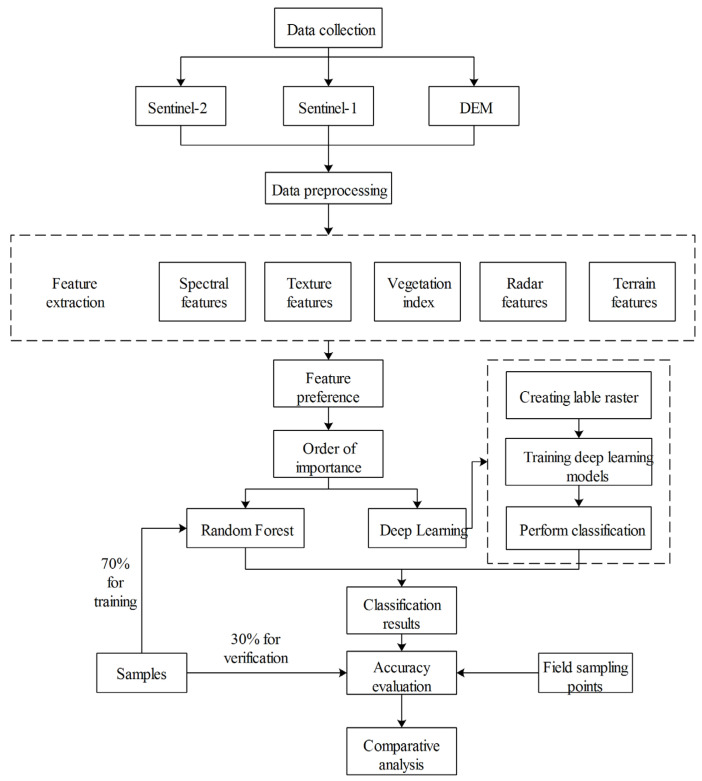
Process of Lily Information Extraction Technology Based on Multi-source Data Fusion.

The technical workflow of this study, as depicted in Figure 2, encompasses the acquisition and preprocessing of Sentinel-2, Sentinel-1, and DEM data. Various spectral features, texture features, vegetation indices, radar features, and terrain features are extracted from the diverse data sources. Feature optimization using the Relief-F algorithm, sorting and grouping the features according to their importance, was combined with deep learning methods to filter the best feature dataset. Random forest and deep learning algorithms were used to classify the images, respectively, and the classification results were evaluated for accuracy and discussed and analyzed to determine the most suitable classification method for the information extraction of lilies in Lanzhou.

### 3.2. Feature Variable Selection

The visible light spectrum is commonly utilized for target extraction, and the unique red-edge band of Sentinel-2A presents a significant advantage in the extraction of agricultural information. In light of this, nine original bands, including red, green, blue, three red-edge bands, a wide near-infrared band, and two shortwave infrared bands from Sentinel-2A, were selected to construct spectral features for experimentation. To enhance crop information extraction and identification, four widely used vegetation indices were chosen. Additionally, six red-edge vegetation indices were constructed based on the red-edge bands of Sentinel-2 images for classification purposes. Building on previous research [22,23], the utilization of the backscattering coefficients (VV and VH polarization) from Sentinel-1 SAR images has been shown to improve classification accuracy. Considering the topographical conditions of crop cultivation areas, variables such as elevation, slope, and aspect were included as features in the classification. Simultaneously, recognizing that texture features can reflect image tone variations and clarity [24], thereby mitigating the “same object, different spectrum” and “different object, same spectrum” phenomena, this study employed the gray-level co-occurrence matrix to extract image texture features. In summary, 48 feature variables, including spectral, vegetation index, texture, radar, and terrain features, were selected for this study, as shown in Table 2.

The gray level co-occurrence matrix (GLCM) is an extensive statistical technique proposed by Haralick and others to process remote sensing data [25,26]. It describes the texture features of an image by calculating the occurrence frequency of pixel pairs with certain numerical and spatial relationships [27]. If texture features are extracted from all bands of Sentinel-2A images separately, this will cause data redundancy. Referring to existing research results [28,29], we performed the principal component analysis (PCA) on Sentinel-2A images to synthesize multiple bands into a few to eliminate extraneous information between bands. The first principal component contains 94.07% of the information in all bands. At the same time, the mean values in four directions (0°, 45°, 90°, and 135°) were selected to calculate eight texture features on three different texture window sizes: 7 × 7, 9 × 9, and 11 × 11 [30].

### 3.3. Feature Preference

Integrating multiple-dimensional feature variables for crop classification can enhance classification accuracy to a certain extent. However, excessively high feature dimensions may introduce low-contributing and weakly correlated features, leading to the curse of dimensionality and data redundancy, ultimately diminishing classification accuracy [31]. Therefore, it is crucial to select the optimal feature set, exclude irrelevant variables, and improve classification accuracy.

Relief is a feature selection algorithm designed for binary classification problems, but it solely considers the classification capability of individual features for neighboring samples, overlooking the interaction between features. To address this limitation, in 1994, Kononenko [32] extended the Relief algorithm and proposed the Relief-F algorithm capable of feature selection for multi-class problems. This algorithm is not only applicable to multi-class classification but also exhibits robustness in handling missing data. Its principle involves selecting a sample *K* from the dataset, calculating the distances between *K* and the nearest neighbors in *q* feature sets of the same class, as well as the distances between *K* and the nearest neighbors in *q* feature sets of different classes. The closer the distance, the higher the correlation, and the greater the weight assigned. This process is iteratively repeated *m* times to determine the weights for each feature [33]. The weight calculation formula is as follows:

$$\begin{array}{l} W(A)=W(A)-\sum_{j=1}^{q}diff(A,K,R_{j})/(m\times q)+\sum_{C\notin class(K)}[\frac{p(C)}{1-p(class(K))}\\ \sum_{j=1}^{q}diff(A,K,N_{j}(C))]/(m\times q) \end{array}$$

where W(A) stands for weight of the feature A; diff(A,K,X) is the difference value between samples K and X on feature A; $R_{j}$ denotes the nearest-neighbor sample in the same sample set as K; $N_{j}(C)$ represents the jth nearest-neighbor sample in the sample set C∉class(N) different from K; P signifies the probability of the category.

### 3.4. Methodology

This study conducted a comparative analysis of the classification performance between the random forest and deep learning methods. A typical representative of the bagging strategy is random forest [34,35]; it is an efficient machine learning algorithm that uses a decision tree as the basic classifier and assembles multiple decision trees together. The construction of the random forest classifier adopts a sampling method that is random and puts back after extraction; a training sample set is created by extracting *N* training samples from the original dataset and then building a decision tree for each training sampling set. Each node of the decision tree randomly selects *k* (*k* ≤ *K*) features from all features *K* throughout the decision tree’s growth. The nodes then utilize information entropy, information gain, or the Gini index to choose the features for node splitting [36]. When a sample is input to be classified, the *N* decision trees have to evaluate the input sample data and their classification characteristics [37], and finally, the classification results of all decision trees are decided by majority voting, and the category with the most votes is the forecast result. The number of decision trees is normally set to *N* = 100 in the parameter settings, and the optimal feature *k* is set to the square root of all feature *K* [38].

The deep learning algorithm used in this study is based on the U-Net model under the TensorFlow framework. It utilizes a set of known feature sample input labels and defined parameters to identify image features based on the spatial and spectral characteristics of the imagery. After training, the model can classify images, enabling the identification of similar features in other images. This model follows a typical convolutional neural network architecture. Prior to training, the TensorFlow model must be initialized, defining the structure of the model, including architecture, patch size, and the number of bands used for training [39]. A patch is a small image provided to the model for training. In this study, the patch size was set to 240, and the number of bands used for training were 5, 14, 24, 36, and 48. After initializing the new model, parameters were set for training in the TensorFlow pixel model. Through multiple iterations, the training process was monitored and evaluated in real time using the TensorBoard visualization tool, tracking metrics such as loss, accuracy, precision, and recall. Combining these results with the classification performance, the optimal parameters were ultimately selected. The parameter settings for deep learning in this study are presented in the Table 3 below.

### 3.5. Accuracy Evaluation

The evaluation of classification results employs the method of confusion matrix [22], utilizing field surveys and randomly selected regions of interest (ROI) from Google Earth as validation samples. The classification outcomes from both random forest and deep learning are compared with the categories of validation samples to construct the confusion matrix. From this matrix, metrics such as producer accuracy (PA), user accuracy (UA), kappa coefficient, and overall accuracy (OA) are computed [40]; producer accuracy and user accuracy are pivotal in determining the extraction of different land-cover information.

## 4. Results and Analysis

### 4.1. Feature Importance Ranking and Grouping

In this paper, the Relief-F algorithm was used to perform feature optimization on 48 features (9 original Sentinel-2A bands, 24 texture features, 10 vegetation index features, 3 terrain features, and 2 polarization features) and rank each feature according to the importance level of each feature, as shown in Table 4. Elevation emerged as the most critical feature, while slope and aspect features also demonstrated high importance, indicating a substantial influence of the terrain on the accuracy of feature classification. Radar features are second only to elevation and slope in the order of importance. Among the vegetation index features, GNDVI exhibited the highest importance, followed by RNDVI, NDVI, RVI, and others. Notably, RNDVI, calculated using the red and red-edge bands, emerged as the most important among the red-edge index features. This shows that adding the red-edge band to the red band can effectively reflect the characteristics of various ground objects.

Both texture and spectral features are of modest importance, with only one 7 × 7 texture feature and no spectral features in the top 12 features and 9 texture features and 2 spectral features in the bottom 12 features. The importance of the correlation features of each window size in texture features is higher than that of other features, indicating that correlation features contribute more to ground object classification compared to other texture features. Additionally, the texture features for each ground object extracted through the GLCM method in a small window demonstrate superior effectiveness. The green light band has the lowest importance among spectral features, followed by the red band, indicating that using only the visible light band has poor classification performance for different ground objects.

We divided the features into five groups according to the order of importance and used deep learning methods for classification. Table 5 shows a trend of increasing and then decreasing feature extraction accuracy in the research area. The accuracy of the classification grows as the number of features increases, and it begins to decline after reaching its peak, which proves that feature optimization may ensure accuracy while reducing the involvement of irrelevant feature variables in classification. The classification accuracy is highest in group 4 with overall accuracy and kappa coefficients of 95.9% and 0.934, respectively. Therefore, group 4 is the optimal feature dataset, and the best features identified in this study number 36, including 3 terrain features, 2 radar features, 7 raw bands, 15 texture features, and 9 vegetation indices.

### 4.2. Classification Results Comparison and Accuracy Evaluation

**Figure 3:**
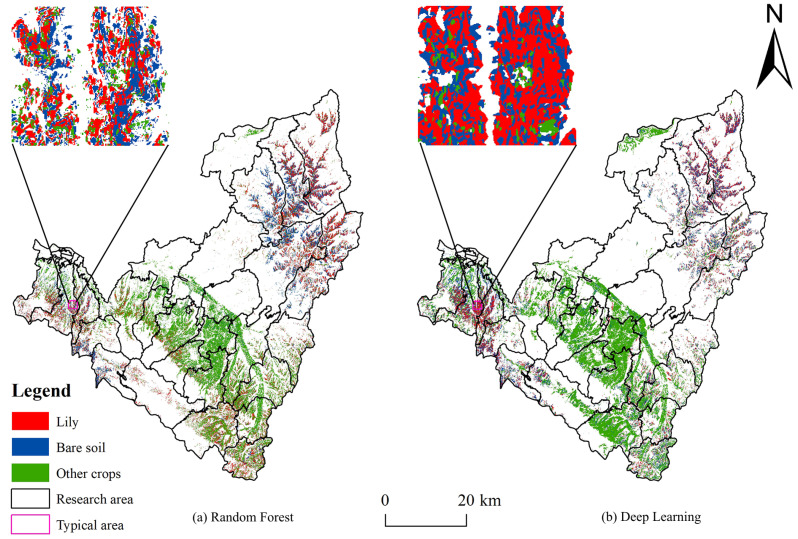
Comparison of Crop Identification and Classification Results in the Study Area.

The optimal feature datasets obtained based on the Relief-F algorithm were classified by random forest and deep learning, respectively, and the spatial planting structure of crops in Qilihe District and Yuzhong County was obtained, as shown in Figure 3. It can be seen that the distribution of the types of ground features classified by the two classification methods is roughly the same, the artificial ground surface is distributed in blocks, and the lily, other crop, and bare lands are distributed in a staggered manner. The extraction results utilizing the random forest algorithm were fragmented, and there is a high incidence of misclassification in some areas, according to a comparison of the classification results of the two approaches. However, the “salt and pepper phenomenon” may be effectively avoided by the classification approach based on deep learning, and the classification effect is good. With a planting area of 137.24 km^2^, the lily-growing region is primarily concentrated in the mountainous regions of Xiguoyuan Township, Weiling Township, and Agan Township in Qilihe District, as well as a portion of the mountainous regions of Shanghuacha Township, Yuanzicha Township, and Zhonglianchuan Township in Yuzhong County.

The feature classification of the study area was performed using the random forest and deep learning methods, respectively, based on the best dataset (36 spectral, textural, exponential, topographic, and radar features), and the confusion matrices calculated based on the comparison of the validation sample categories with the classification results are shown in Table 6 and Table 7. Among them, overall accuracy was 94.6% and 95.9%, respectively, and the kappa coefficients were 0.911 and 0.934, respectively. It can be seen that the performance of the random forest classification method in extracting lily information is not as good as the deep learning method in this article. The user accuracy and producer accuracy of the random-forest-extracted lilies are 87.3% and 94.8%, respectively, while the user accuracy and producer accuracy of the deep-learning-extracted lilies are 93.7% and 91.6%, respectively, as shown in Table 6 and Table 7.

In the confusion matrices for random forest and deep learning classification, 204 and 75 lily image pixels were classified as other crops, while 51 and 65 other crop image pixels were classified as lily, respectively. In addition, there were several image pixel misclassifications in lilies, other crops, and bare soil. In random forest, 18 bare soil image pixels were categorized as lily and 31 bare soil image pixels as other crops; in deep learning, 16 bare soil image pixels were classified as lily and 45 bare soil image pixels as other crops. As other crops, bare soil, and lilies have comparable topographic features, this leads to more misclassifications of lilies with other crops and bare soil, which reduces the classification accuracy of lilies.

## 5. Discussions

In this paper, 48 features such as spectrum, texture, and red-edge index were chosen to extract lily planting information based on the planting conditions of lilies in the study area and using Sentinel-1, Sentinel-2, and DEM as data sources, avoiding the problem of the low classification accuracy of a single feature. However, too many characteristics are prone to data redundancy, which reduces the classification effect. As a result, the Relief-F algorithm is used to sort and group the importance of features, and the deep learning approach is utilized to select the ideal feature group, with a total of 36 features. Deep learning classification with only terrain and radar features is poor, with an overall accuracy of only 75.7%; the overall accuracy increases to 87.2% with the addition of partial vegetation indexes and red-edge indexes, and the overall accuracy increases to 90.5% with the addition of the more important texture features on top of the terrain, radar, and partial index features. As the number of features rises, so does the classification accuracy. When all features are classified, the accuracy falls, the most accurate feature group is chosen as the optimal feature dataset, and the 12 features with a lower-importance ranking are eliminated (ranked 37–48 in Table 4). The producer accuracy and user accuracy of diverse ground objects are both greater than 76%, according to the random forest and deep learning classification results based on the optimal feature dataset. Among them, the producer accuracy and user accuracy of lilies are relatively excellent, both exceeding 87%. The overall accuracy of the deep learning classification algorithm for the optimal feature dataset is 95.9%, with a kappa coefficient of 0.934. The overall accuracy and kappa coefficients have increased by 1.3% and 0.023, respectively, as compared to the random forest classification approach. This indicates that the combination of the Relief-F and deep learning classification methods performs well in extracting lily planting information in Lanzhou, which can meet the requirements of lily planting structure extraction and spatial distribution monitoring in Lanzhou.

The deep learning classification method based on multi-source data in this article produced satisfactory results. However, deep learning approaches necessitate a huge quantity of data to train the model, and parameter adjustments have a certain degree of subjectivity and exploratory nature. As a result, further research is needed in parameter adjustment and training techniques. Feature optimization can improve classification accuracy, but different feature optimization methods are based on different models and principles, and the results obtained by a single feature optimization method may be one-sided, so it is necessary to consider combining multiple feature optimization methods to obtain the best feature. In addition, this paper is based on single-phase remote sensing image information extraction. In most cases, the classification accuracy of multi-temporal is significantly higher than that of single-phase. In the subsequent research, images with obvious phenological differences in different periods are selected for classification as far as possible.

## 6. Conclusions

In this study, we extracted spectral, texture, vegetation index, red-edge index, topography, and radar features from Sentinel-1, Sentinel-2A, and DEM data to construct a multidimensional feature dataset, excluded irrelevant variables using the Relief-F algorithm, selected the optimal feature dataset, and extracted the lily planting structure in Qilihe District and Yuzhong County using a deep learning method and compared it with the random forest classification method for comparison. The main findings are as follows:

(1) Using the Relief-F algorithm for feature selection and feature importance ranking, deep learning classification revealed that accuracy increased initially and subsequently decreased, achieving the best accuracy when the number of features was 36. According to the importance ranking of features, it can be seen that, among the topographic features, elevation has the greatest contribution in feature classification, radar features are only followed by elevation and slope, texture features and spectral features have lower contributions, and the addition of red light bands to red-edge bands can effectively reflect the characteristics of each feature.
(2) Deep learning classification accuracy is higher than random forest classification accuracy under the same feature dimension, with overall accuracy of 95.9% and a kappa coefficient of 0.934, which can inhibit the generation of the “pepper and salt phenomenon” and more accurately extract the planting structure information of lilies in the study area.
(3) The lily planting area extracted based on multi-source data images is 137.24 km^2^, mainly concentrated in Xiguoyuan Township in Qilihe District and in Shanghuacha Township and Yuanzicha Township in Yuzhong County.

The deep learning method based on multi-source data exhibits promising performance in extracting structural information concerning lily cultivation. Looking ahead, future work could delve into optimizing the fusion of unmanned aerial vehicle (UAV) data with other data sources, improving algorithms for more accurate feature extraction and exploring the integration of advanced sensing technologies to extract more accurate crop areas. In addition, the method can be used not only for agricultural production management but also extended to other fields such as food security monitoring, land use planning, and environmental protection. As an effective means of digital agriculture, the method is expected to promote the development of agriculture in a more efficient and sustainable direction.

## Figures and Tables

**Table 1:** Training samples and validation samples.

Ground Object Code	Ground Object Name	Training Sample Size	Verification Sample Size
0	Lily	244	105
1	Bare soil	205	88
2	Other crops	226	97
3	Natural land	175	75
4	Artificial land	164	71
Total		1014	436

**Table 2:** Overview of Feature Variable Sets.

Feature Variable	Abbreviation	Explanation or Calculation Formula
Spectral Index	Band	B2, B3, B4, B5, B6, B7, B8, B11, B12
Vegetation Index	NDVI	(B8−B4)/(B8 + B4)
	DVI	B8-B4
	GNDVI	(B8−B3)/(B8 + B3)
	RVI	B8/B4
	NDVIre1	(B8−B5)/(B8 + B5)
	NDVIre2	(B8−B6)/(B8 + B6)
	NDVIew3	(B8−B7)/(B8 + B7)
	NDre1	(B6−B5)/(B6 + B5)
	NDre2	(B7−B5)/(B7 + B5)
	RNDVI	(B5−B4)/(B5 + B4)
Texture Features	MEA	Mean
	VAR	Variance
	ENT	Entropy
	COR	Correlation
	CON	Contrast
	DIS	Dissimilarity
	HOM	Homogeneity
	ASM	Angular Second Moment
Radar Signature	VV	VV polarization backscatter coefficient
	VH	VH polarization backscatter coefficient
Terrain Features	DEM	Elevation
	Slope	Slope of ground
	Aspect	The direction the slope faces

**Table 3:** The best parameters for model training.

Parameter Name	Number of Epochs	Patches per Epoch	Patch Sampling Rate	Blur Distance	Class Weight	Loss Weight
Parameter Value	25	300	16	0–25	4.5	1.2

**Table 4:** Feature importance ranking.

Ranking	Feature Name	Importance	Ranking	Feature Name	Importance
1	elevation	0.032441	37	B4	0.000948
2	Slope	0.016145	38	B3	0.000885
3	VH	0.012775	39	NDVIRE3	0.000810
4	VV	0.012379	40	DIS-11	0.000220
5	Aspect	0.011868	41	DIS-7	0.000159
6	COR-7	0.009081	42	DIS-9	0.000122
7	GNDVI	0.009016	43	CON-11	0.000014
8	RNDVI	0.008818	44	VAR-11	0.000013
9	NDVI	0.008671	45	CON-7	0.000006
10	RVI	0.006160	46	VAR-9	0.000005
11	NDRE1	0.005591	47	CON-9	0.000005
12	NDRE2	0.005559	48	VAR-7	0.000002

**Table 5:** Grouping.

Group	Grouping Threshold	Number of Features	Classification Accuracy (%)
1	0.01	5	75.7
2	0.005	14	87.2
3	0.003	24	90.5
4	0.001	36	95.9
5	0.000002	48	92.3

**Table 6:** Confusion matrix for random forest methods.

Ground Object Code	0	1	2	3	4	Total	UA (%)
0	1766	25	204	29	0	1821	87.3
1	18	939	31	7	12	1201	93.3
2	51	95	1397	34	1	1877	88.5
3	26	84	195	9649	18	9907	96.8
4	2	19	10	42	2095	2043	96.6
Total	1863	1162	1837	9761	2126		
PA (%)	94.8	80.8	76.1	98.9	98.5		

**Table 7:** Confusion matrix for deep learning methods.

Ground Object Code	0	1	2	3	4	Total	UA (%)
0	1707	37	75	2	0	1821	93.7
1	16	1060	45	78	2	1201	88.3
2	65	43	1657	65	47	1877	88.3
3	74	13	53	9616	51	9907	98.1
4	1	9	7	0	2026	2043	99.2
Total	1863	1162	1837	9761	2126		
PA (%)	91.6	91.2	90.2	98.5	95.3		

Note: Codes 0–4 represent the names of different feature types as shown in Table 1.

## Data Availability

The new data created in this study are available on request.
